# Histone H2A and H2B Are Monoubiquitinated at AID-Targeted Loci

**DOI:** 10.1371/journal.pone.0011641

**Published:** 2010-07-16

**Authors:** Glen M. Borchert, Nathaniel W. Holton, Kevin A. Edwards, Laura A. Vogel, Erik D. Larson

**Affiliations:** School of Biological Sciences, Illinois State University, Normal, Illinois, United States of America; University of Minnesota, United States of America

## Abstract

**Background:**

Somatic hypermutation introduces base substitutions into the rearranged and expressed immunoglobulin (Ig) variable regions to promote immunity. This pathway requires and is initiated by the Activation Induced Deaminase (AID) protein, which deaminates cytidine to produce uracils and UG mismatches at the Ig genes. Subsequent processing of uracil by mismatch repair and base excision repair factors contributes to mutagenesis. While selective for certain genomic targets, the chromatin modifications which distinguish hypermutating from non-hypermutating loci are not defined.

**Methodology/Principal Findings:**

Here, we show that AID-targeted loci in mammalian B cells contain ubiquitinated chromatin. Chromatin immunoprecipitation (ChIP) analysis of a constitutively hypermutating Burkitt's B cell line, Ramos, revealed the presence of monoubiquitinated forms of both histone H2A and H2B at two AID-associated loci, but not at control loci which are expressed but not hypermutated. Similar analysis using LPS activated primary murine splenocytes showed enrichment of the expressed V_H_ and Sγ3 switch regions upon ChIP with antibody specific to AID and to monoubiquitinated H2A and H2B. In the mechanism of mammalian hypermutation, AID may interact with ubiquitinated chromatin because confocal immunofluorescence microscopy visualized AID colocalized with monoubiquitinated H2B within discrete nuclear foci.

**Conclusions/Significance:**

Our results indicate that monoubiquitinated histones accompany active somatic hypermutation, revealing part of the histone code marking AID-targeted loci. This expands the current view of the chromatin state during hypermutation by identifying a specific nucleosome architecture associated with somatic hypermutation.

## Introduction

The immunoglobulin (Ig) genes in activated B cells are diversified by somatic hypermutation and class switch recombination to promote immunity. Somatic hypermutation introduces point mutations into the rearranged and expressed Ig variable regions while class switch recombination coordinates the exchange of one Ig constant region for a downstream region, deleting the intervening DNA. Both pathways require the Activation Induced cytidine Deaminase (AID) protein [1], [2]. AID is active on transcribed DNA and functions to convert single-stranded cytidines into uracil and uracil-guanine mismatches (reviewed in [3], [4]). Canonical mismatch repair and base excision repair are able correct genomic uracil [5]–[7], but in somatic hypermutation these normally faithful repair pathways become mutagenic [8]. The diversion to mutagenesis is promoted by the synthesis activities of low-fidelity DNA polymerases, such as polymerase eta (reviewed by [9], [10]), and their participation in hypermutation may be regulated by PCNA monoubiquitination (reviewed by [11]). While hypermutation is largely confined to the rearranged and expressed Ig genes, other B cell loci are prone to aberrant hypermutation, leading to lymphoma [12], [13]. The molecular mechanisms responsible for targeting AID to certain sites in the genome and the regulation of subsequent mutagenesis have not been established.

Monoubiquitination pathways may be important for regulating Ig gene diversification. DT40 B cells disrupted for PCNA monoubiquitination by a K164R substitution showed decreased AID-initiated Ig gene diversification [14]. Similarly, PCNA K164R knock-in mice had an altered spectrum of Ig gene mutagenesis, paralleling mismatch repair defective mice and suggesting that monoubiquitination of PCNA influences uracil repair outcomes in mammals [15], [16]. PCNA modification may be facilitated in part by the RAD6 pathway because inactivation of a RAD6 associating E3 ligase, RAD18, in DT40 B cells resulted in reduced levels of PCNA monoubiquitination and decreases in hypermutation [14], [17], however residual PCNA monoubiquitination in *rad18* mutant DT40 suggests the involvement of more than one E3 ligase [18]. Further evidence for protein ubiquitinations in Ig gene diversification pathways comes from recent studies on the RNF8 and RNF168 E3 ligases. Silencing of these two proteins decreased class switch recombination efficiency in a murine model B cell line [19], and RNF168 was identified as the RIDDLE syndrome protein, a disease characterized by immunodeficiencies and DNA repair defects [20]. Recently, RNF8 knock-out mice were shown to have impaired class switch recombination and defects in DNA damage responses [21], [22], further connecting histone monoubiquitination with Ig gene diversification. Together, it is likely that multiple proteins are monoubiquitinated to promote Ig gene diversification and hypermutation specific histone E3 ligases could serve as signals for locus-specific mutagenesis.

In light of the DNA repair machinery-driven nature of hypermutation at the Ig loci, characterized roles for chromatin monoubiquitination in facilitating normal DNA repair throughout the genome (reviewed by [23]), and the involvement of RNF8 in class switching [19], [21]–[22], we speculated that histone monoubiquitination could accompany hypermutation. We therefore assayed the nucleosomes at AID-targeted and actively hypermutating loci for ubiquitin modification. Here, we show by Chromatin Immunoprecipitations (ChIPs) that both histone H2A and H2B are monoubiquitinated at two different AID-targeted loci in the constitutively hypermutating B cell line Ramos. ChIP analysis of primary LPS activated murine splenocytes showed similar levels of enrichments for hypermutating template upon immunoprecipitation with antibody specific to AID, pol II, and monoubiquitinated forms of H2A and H2B. Furthermore, we find by immunofluorescence microscopy that AID colocalizes with monoubiquitinated H2B in discrete nuclear foci, suggesting that both are concomitant with hypermutation. Our results identify a considerable and previously uncharacterized chromatin modification associated with AID-induced somatic hypermutation.

## Results

### AID and hypermutation target two discrete loci in Ramos

**Figure 1:**
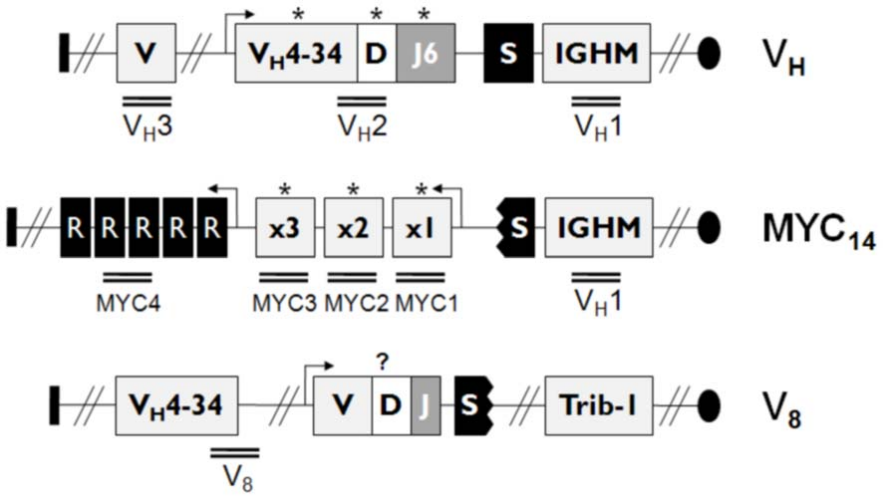
Diagram of the IgH loci in Ramos Burkitt's lymphoma. A reciprocal translocation exchanging the ends of chromosomes 8 and 14 in Ramos resulted in the formation of *MYC_14_* and *V_8_*. The chromosome 14 break point occurred within the heavy chain (IgH) switch region (indicated by jagged edges) just 5′ of the µ constant exon (IgHM) while the chromosome 8 breakpoint occurred in a *c-MYC* allele promoter. (R), silent, non-coding RNA; ×1, ×2, ×3, *c-MYC* exons. Arrows indicate transcriptional direction. Double bar denotes amplicon location. Primers were designed to amplify the IgH constant region mu (V_H_1), the functional heavy chain V_H_4-34/D/J6 rearrangement (V_H_2), and an upstream non-rearranged V-region sequence (V_H_3). Chromosome 8 primer sets amplify three *c-MYC* exons (MYC1–3), and a sequence 50 kb 5′ of MYC3 (*MYC4*). One primer set was designed for the non-expressed unrearranged V_H_ 4–34 on chromosome 8 (*V_8_*).

Ramos is a human Burkitt's lymphoma cell line with both functional and non-functionally rearranged Ig heavy chain (IgH) loci. One IgH allele contains a productive VDJ rearrangement (re-*V_H_*), which is a gene that is expressed and constitutively hypermutated [24]. The other IgH allele has participated in a reciprocal translocation between the ends of chromosomes 8 and 14, replacing the antibody variable region on chromosome 14 with an intact *c-MYC* allele from chromosome 8 (*MYC_14_*), and this locus is also hypermutated [25]. The partially rearranged V region on chromosome 8 and the mu constant region downstream of re-*V_H_* are not diversified by hypermutation. Using EST data from the NCBI database, and previously reported *c-MYC* sequences [25], we constructed a map of the rearranged and expressed heavy chain loci in Ramos (Fig. 1). The documented constitutive hypermutation of Ramos re-*V_H_* and *MYC_14_* predict that AID selectively targets these two distinct loci for deamination.

**Figure 2:**
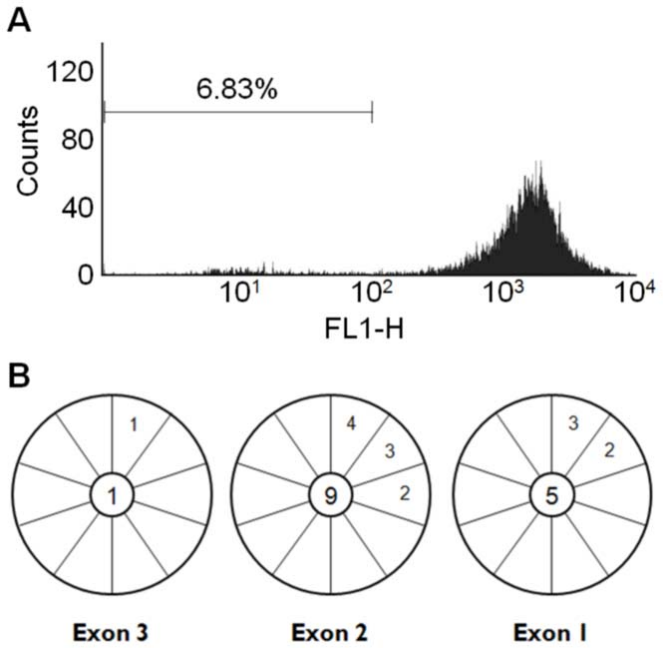
Ramos Burkitt's lymphoma cells constitutively hypermutate at distinct genomic loci. A. Flow cytometry measure of loss of surface IgM (sIgM) display over time for cultured Ramos cells. sIgM was detected using FITC conjugated antibody specific for human IgM (FL1-H). Ramos cells were continuously cultured for 90 days after which 10,000 cells were analyzed. B. Sequence analysis of the translocated and expressed *MYC_14_*. Each pie wedge represents a unique sequencing read with the number of mutations identified in individual reads indicated. The total number of mutations identified in 10 unique sequence reads corresponding to individual exons is shown in the central circles.

We first established active somatic hypermutation of the re-*V_H_* and *MYC_14_* in our Ramos subclone using standard surface IgM loss assays and direct sequencing of *MYC_14_*. Constitutive sequence diversification of the expressed IgM locus results in mutations that inactivate surface display, providing a marker for hypermutation rate in Ramos. Starting from a single Ramos sIgM positive isolate, 90 days of continuous culture resulted in 6.83% sIgM loss, as measured by FACS with FITC-conjugated anti-IgM antibody (Fig. 2A). This compares well with the sIgM loss rate reported for Ramos, showing 4% sIgM loss for after 4 weeks of in vitro culture [26], and ∼17% loss after 6 months [24]. We conclude that the re-*V_H_* is actively hypermutated. To ensure ongoing hypermutation of the other IgH locus, individual expressed *MYC_14_* clones of from our Ramos population were sequenced, and 20% of our sequence reads contained one or more novel mutations (6/30, mean length = 748 nt) (Fig. 2B). Only the translocated *c-MYC* is expressed in Ramos, and *MYC_14_* contains a single nucleotide polymorphism A523T [25], distinguishing it from the non-translocated allele. This base substitution was present in all sequence reads we obtained. The *MYC_14_* point mutations we identified are novel, and do not correspond to previously identified *c-MYC* hypermutations [25]. We conclude that both the re-*V_H_* and *MYC_14_*_ in_ our Ramos subclone are expressed and subject to hypermutation, as expected.

**Figure 3:**
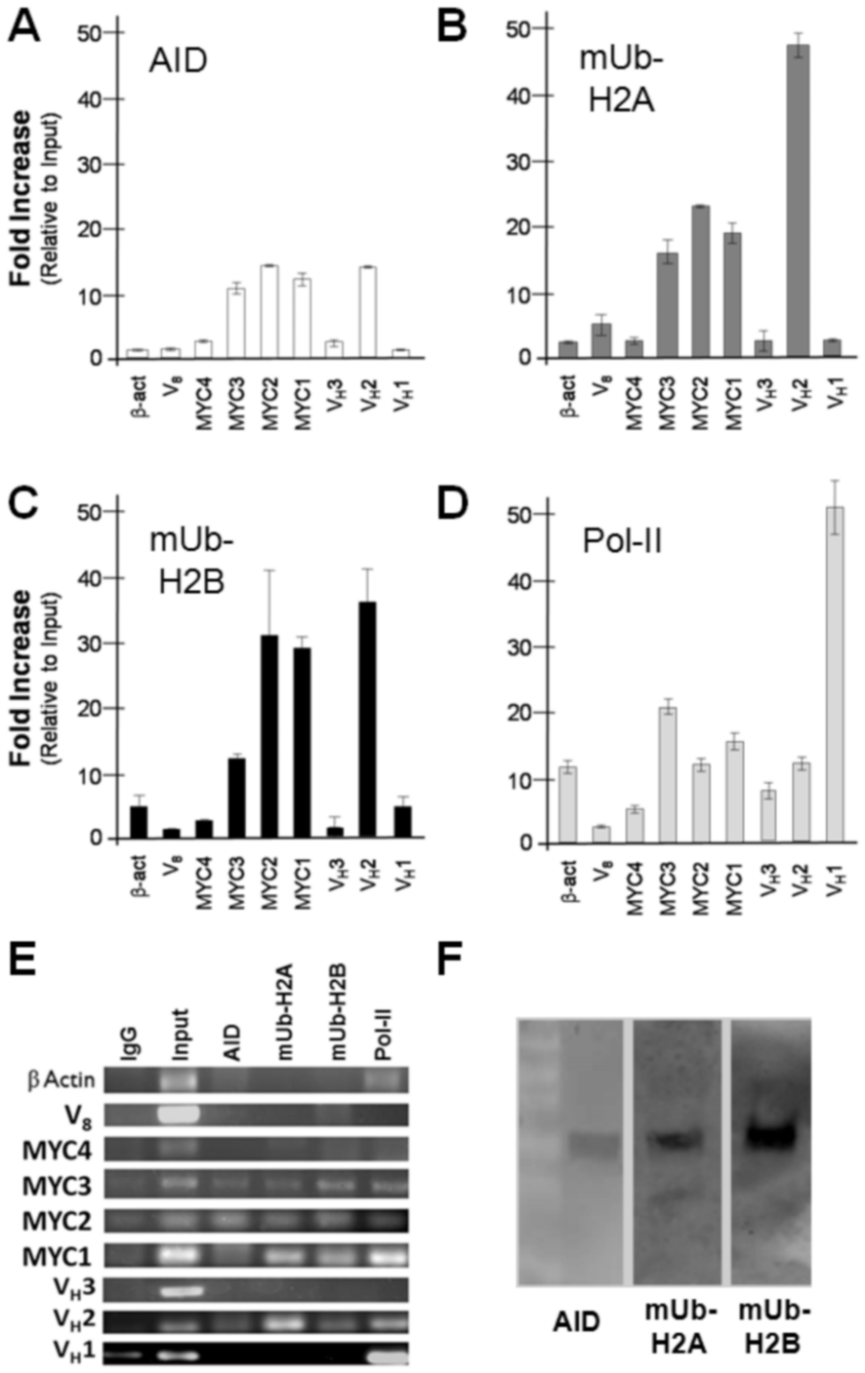
ChIPs identify mUb-H2A and mUb-H2B at Ramos hypermutating *loci*. A. Normalized template enrichment upon anti-AID ChIP. Template obtained from AID-IP was amplified and analyzed by qPCR using primer sets diagramed in Figure 1, above. Results were normalized to non-specific IgG IP and relative to input. Each bar indicates the mean of the values obtained from six amplifications (two separate experiments analyzed in triplicate) with standard deviation. B. Normalized template enrichment upon mUb-H2A ChIP. C. Normalized template enrichment upon mUb-H2B ChIP. D. Normalized template enrichment upon pol II ChIP. Enrichments were analyzed by qPCR as in Figure 3A. E. Ethidium bromide stained gel of traditional PCR amplifications from ChIP template. F. Antibodies used to ChIP mub-H2A and mub-H2B and AID are specific for monoubiquitinated H2A, H2B and AID protein. Western blot analysis of endogenous AID (24 kDa), mUb-H2A (27 kDa) and mUb-H2B (27 kDa) in Ramos cell lysates. Visible on the AID blot, the white negative stained bands of the protein marker correspond to 55, 40, 36, 25, 15 and 10 kilodaltons (top to bottom).

AID activity is required for initiating somatic hypermutation, predicting that the protein should be in physical contact with both the Ramos re-*V_H_* and *c-MYC* genes. To test this, we used chromatin immunoprecipitations (ChIPs) to test for AID association with hypermutating loci in Ramos. ChIPs rely on specific antibodies to precipitate crosslinked protein-DNA complexes, followed by quantitative PCR (qPCR) analysis of the enriched genomic templates. Primer sets were designed to amplify templates immunoprecipitated from Ramos cells corresponding to the re-*V_H_* locus (V_H_ 1–3), the *c-MYC* gene (exons 1–3), and several non-hypermutating control loci (Fig. 1). In chromatin prepared from Ramos cells, the normalized enrichment of the re-*V_H_* using anti-AID antibody was 13.8-fold (Fig.3A). Likewise, AID ChIP enriched for exons 1–3 of *c-MYC* (11.9 fold, 14.0 fold and 10.8-fold, respectively) (Fig. 3A). AID ChIP did not enrich for any of the other loci tested, including a distal downstream (50 kb) region of *MYC_14_* (*MYC4*), the Cmµ constant region (V_H_ *-Cµ*), the non-productively rearranged heavy chain V region on chromosome 8 (*V_8_*), and the β actin gene (Fig. 3A). To further test hypermutation specificity ChIPs with PCNA and pol eta antibodies enriched for the re-*V_H_* (Fig. S1), and AID antibody failed to enrich for the *c-MYC* locus in AID negative HEK293 and A549 cells (Fig. S2), and westerns with this same antibody did not detect AID in HEK293 extracts, but identified a single 24 kDa band in Ramos, as expected (Fig. S3). In addition, IPs using this AID antibody and Ramos extract precipitated native AID, as detected by western with an independent AID antibody (Fig. S4).

Primers designed to amplify *MYC_14_* could potentially amplify the other *c-MYC* allele if precipitated during ChIPs; however, only the translocated *c-MYC* contains the single nucleotide polymorphism A523T [25]. Further, ChIP results are suggestive of AID association with *MYC_14_* because AID tracks with pol II to deaminate transcribed DNA (reviewed by [27]) and the non-translocated allele of *c-MYC* is not expressed ([25] yet pol II ChIPs enriched for exons 1–3 *c-MYC*. Most importantly, 100% (8/8) of different sequence reads from PCR product amplified from anti-AID ChIPed template corresponded to the translocated *MYC_14_* allele (not shown). Together, our results indicate that AID is physically associated with the Ramos re*-V_H_* and with exons 1–3 of *MYC_14_*, supporting the model that both loci are subject to AID-initiated somatic hypermutation.

### The chromatin at the re-*V_H_* and *c-MYC* gene in Ramos B cells is monoubiquitinated

Having confirmed AID associations at both re-*V_H_* and c-*MYC* in our Ramos subculture, we next examined the chromatin state at these loci to identify histone markers of hypermutation. Based on roles for histone ubiquitination in normal DNA repair [23] and apparent involvement of RNF8/168 ubiquitin E3 ligases in class switch recombination [19]–[22], we focused our analysis on ubiquitin-modified chromatin. We used ChIPs and antibodies specific to monoubiquitinated histones to ask if the *re-V_H_* and *MYC_14_* loci are modified by ubiquitination. Analysis of qPCR following mUb-H2A ChIP of Ramos chromatin showed a 48.3 fold normalized enrichment of the re-*V_H_*. Exons 1–3 of *c-MYC* were also enriched, showing 18.4 fold, 23.0 fold and 16.6 fold enrichments respectively (Fig. 3B). Similar to the mUb-H2A ChIP, qPCR analysis following mUb-H2B ChIP of Ramos chromatin showed 37.6-fold normalized enrichment of the re-*V_H_* and exons 1, 2, and 3 of *c-MYC* showed 29.3 fold, 32.3-fold and 12.9-fold enrichments, respectively (Fig. 3C). In contrast, neither the *V_H_*-*Cµ*, *V_8_*, *β-actin* gene, nor *MYC4* were enriched by mUb-H2A or mUb-H2B ChIP (Figs. 3B, C). Also, mUb-H2A and mUb-H2B ChIPs with AID negative HEK293 and A549 cells did not enrich for the *c-MYC* loci (Fig. S2). We conclude that the re-*V_H_* and *c-MYC* loci in Ramos are occupied by nucleosomes that contain monoubiquitinated forms of H2A and H2B.

Previous studies connect histone monoubiquitination with transcriptional silencing and/or polymerase elongation (reviewed by [28]) so we next asked if histone ubiquitination is a broad consequence of high RNA polymerase II (pol II) driven expression of the heavy chain loci in Ramos, or associated with mutagenesis. ChIPs using RNA pol II antibody and Ramos chromatin showed comparable enrichment via normalized qPCR for the re-*V_H_*, exons 1–3 of *c-MYC*, *β-actin*, and *V_H_*-*Cµ*. We did not observe enrichment of the unrearranged V_H_ locus (*V_8_*) or the region downstream of *MYC_14_*, *MYC4*, which was anticipated because these regions are not expressed. In contrast, *β-actin*, and *V_H_-Cµ* were all enriched by pol II ChIPs (Fig. 3D), but not after ChIP using monoubiquitinated histone antibody (Figs. 3B, C). These results mirror our AID analyses, and suggest that pol II-dependent gene expression alone is insufficient for establishing sustained chromatin monoubiquitination in Ramos.

If monoubiquitinated chromatin is associated with Ig gene mutagenesis, we also anticipate other hypermutation factors will be associated with these same loci. AID-induced uracil is processed by mismatch repair factors at the Ig loci, and substitutions are introduced opposite A and T bases through PCNA-dependent recruitment of pol eta synthesis in place of pol delta (reviewed by [11]). ChIP analysis suggests that the re-*V_H_*, but not the unrearranged allele (*V_8_*), is in physical contact with PCNA and pol eta (Fig. S1), supporting the model that the monoubiquitinated loci we identify here also harbor proteins known to support mutagenesis in response to uracil in DNA. The qPCR enrichments obtained for Ramos ChIPs were independently verified by qualitative, traditional PCR amplification and ethidium bromide visualization (Fig. 3E) and westerns verified specificity for AID and monoubiquitinated histone antibodies (Fig. 3F).

### The chromatin of Ig genes in activated primary murine B cells is ubiquitinated

**Figure 4:**
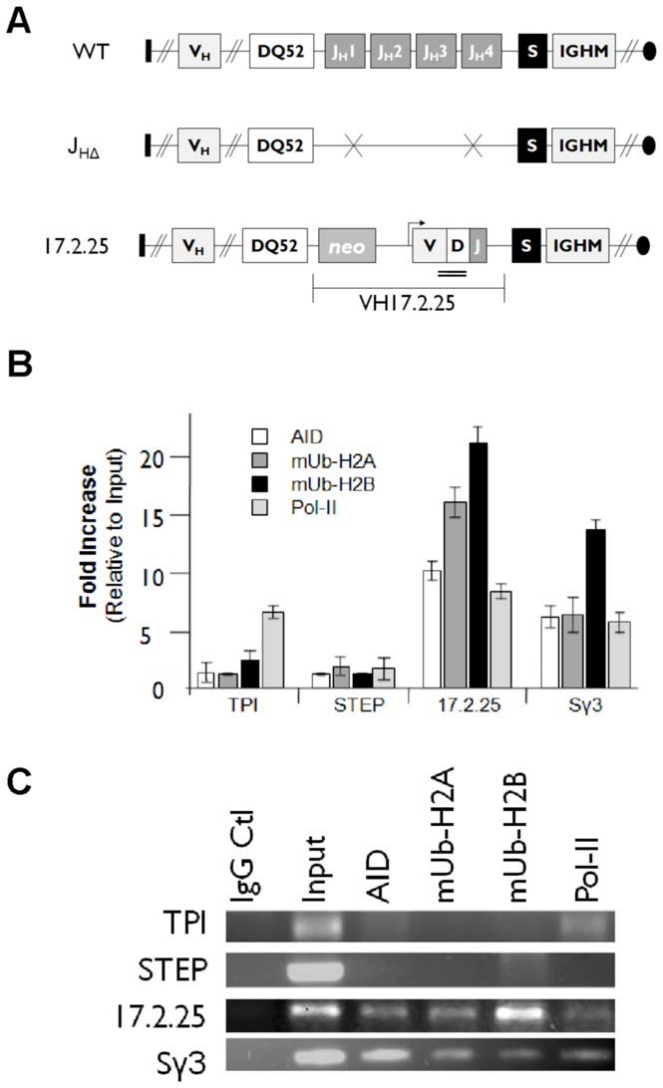
mUb-H2A and mUb-H2B associate with hypermutation in activated mouse primary B cells. A. Cartoon depicting the engineered rearranged heavy chain allele sequences in quasi-monoclonal mouse [29]. The rearranged allele (17.2.25) is compared to a normal heavy chain arrangement (wt). The 17.2.25, allele formed by the targeted replacement of the joining cluster with a defined V(D)J rearrangement. J_HΔ_, allele formed by the targeted deletion of the joining cluster. S, Sγ3 switch region. IGHM, µ constant exon. V_H_ additional 5′ variable sequence segments. DQ52, diversity sequence segment immediately 5′ of the joining sequence cluster. Arrows indicate transcriptional direction. Double bar denotes amplicon location. B. ChIPs identify monoubiquitinated histones with hypermutation. Murine splenocytes were cultured for 72 hours with LPS, crosslinked, and then subjected to ChIP analysis with anti-mUb-H2A and anti-mUb-H2B, and anti-AID antibody. Normalized enrichment of the 17.2.25, Sγ3, triose phosphate isomerase (TPI) and neural-specific striatum-enriched protein-tyrosine phosphatase (STEP) loci from qPCR are shown relative to input DNA. C. Ethidium bromide visualization of traditional PCRs with primer sets corresponding to the qPCRs.

It is possible that chromatin monoubiquitination is a unique feature of Ramos hypermutation. To address this, we tested histone monoubiquitination of an AID-targeted locus in LPS activated murine B cells. Typically, hypermutation is difficult to quantify by PCR in primary cells because of the sequence variability inherent to the rearranged Ig genes. To overcome this technical limitation, we used a quasi-monoclonal mouse, which was engineered to contain a functional IgH chain allele generated by targeted replacement of heavy chain locus variable region with a defined VDJ rearrangement (17.2.25) [29] (Fig. 4A). The 17.2.25 VDJ region in these mice is subject to diversification by somatic hypermutation [30].

Primary splenocytes were isolated from six healthy quasi-monoclonal mice and cultured with lipopolysaccharide (LPS) to activate primary B cells. ELISAs for secreted IgG confirmed activation of Ig gene diversification (data not shown). In chromatin prepared from these activated splenocytes, normalized enrichment of 17.2.25 after ChIP with mUb-H2A and mUb-H2B antibody showed robust enrichment, 16.2-fold and 21.8-fold for each antibody, respectively (Fig. 4B). Similarly, pulldowns using these same antibodies were also enriched for the Sγ3 switch region (Fig. 4B). AID appears to target the 17.2.25 and Sγ3 loci after activation because anti-AID ChIPs resulted in 10.4 and 7.1 fold enrichments of these regions respectively (Figs. 4B, C). The murine triose phosphate isomerase gene (TPI) and unexpressed neural specific gene (STEP) were used as controls, and only the TPI locus showed modest enrichment (6.9-fold) after pol II ChIP, and neither locus was precipitated upon IP with anti-AID, anti-mUb-H2A, or anti-mUb-H2B. Due to the limited antibody repertoire encoded by the quasi-monoclonal mice, selective pressure for Ig gene diversity is high, causing both hypermutation and frequent V gene replacements [30]. Because it is unlikely that 17.2.25 gene rearrangements would be amplified by our primer sets, enrichments we obtained for the 17.2.25 locus after AID ChIP (Fig. 4B) are likely to be an under-representation of the actual level of AID association. Regardless, we conclude that the 17.2.25 and Sγ3 loci from primary LPS activated quasi-monoclonal murine splenocytes are expressed by RNA pol II, targeted by AID, and occupied by monoubiquitinated histone H2A and histone H2B.

### AID and mUb-H2B colocalize in discrete nuclear foci

**Figure 5:**
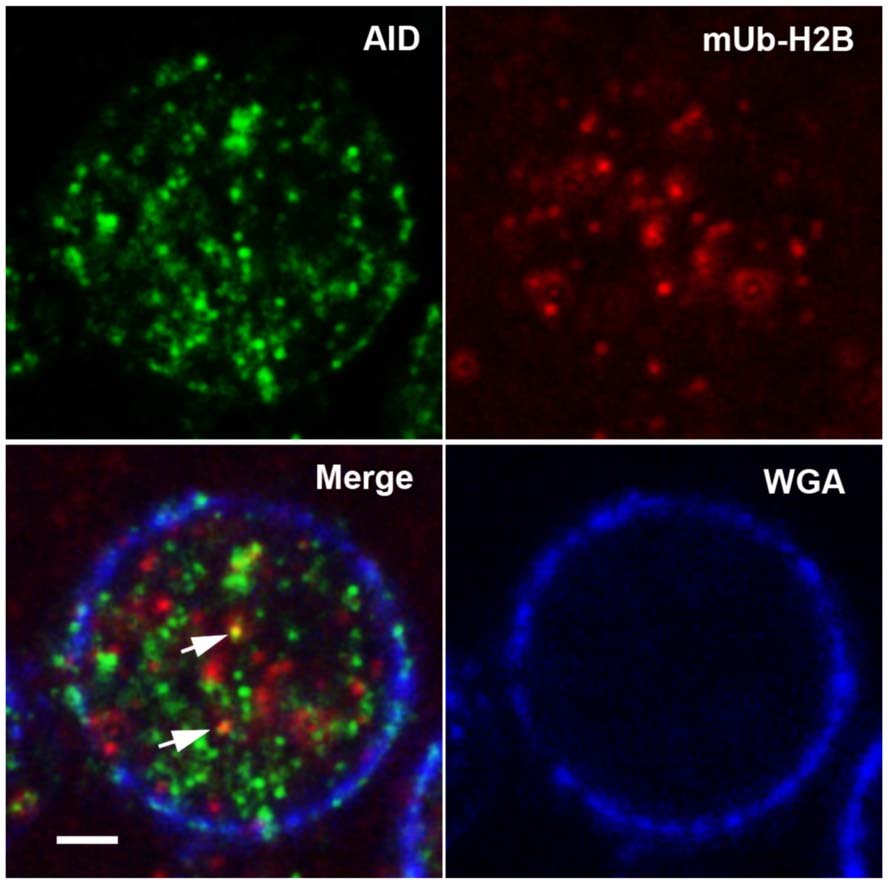
AID and monoubiquitinated H2B colocalize in discrete nuclear foci. Representative immunofluorescence confocal microscopy images of Ramos cells stained for AID and mUb-H2B. Ramos cells were incubated with rabbit antibody specific to AID and murine mUb-H2B antibody followed by Alexa-488 conjugated rabbit secondary and Alexa-555 conjugated murine secondary antibody. Upper left, Ramos cells imaged with 488 nm filter. Upper right, Ramos cells imaged with 555 nm filter. Lower left, merged image with white scale bar (2 microns). Lower right nuclear envelope imaged using wheat germ agglutinin (Blue). Arrows point to colocalizations of AID and mUb-H2B.

ChIPs examine total cellular populations, and hypermutation events may occur transiently and at unsynchronized time points. Therefore, we used immunofluorescence confocal microscopy to ask if AID and ubiquitinated chromatin co-occupy the DNA. Both mUb-H2B and AID antibody produced clear robust signals in Ramos cell preparations. Figure 5 shows a representative image of AID and mUb-H2B foci in Ramos, and overlay of these images showed clear colocalization of AID and mUb-H2B signal in discrete nuclear foci. We also found multiple examples where more than one AID and monoubiquitinated H2B are colocalized within a single cell nucleus (Fig. S5), supporting the notion that at least two and possibly more loci are diversified by somatic hypermutation in Ramos cells. We observe multiple AID foci in individual Ramos cells, highly consistent with the behavior of endogenous AID [31]–[33]. It is possible that non-Ig genes are bound and deaminated by AID in Ramos. Antibodies to mUb-H2A and mUb-H2B antibody are specific for the monoubiquitinated forms of histones, and our westerns probing Ramos whole cell extract clearly show single bands at ∼25–30 kDa (Fig. 3F) corresponding to the size of a single histone ubiquitination (∼28 kDa). Even though mUb-H2A, and mUb-H2B antibodies were competent for western analyses (Fig. 3F) and ChIPs (Figs. 3 and 4), we were unable to clearly detect mUb-H2A by immunofluorescence microscopy. Nevertheless, our imaging results suggest that AID is spatially and temporally associated with ubiquitinated nucleosomes.

## Discussion

Our results show that genomic loci that are targeted for somatic hypermutation are also occupied by monoubiquitinated chromatin. Histone H2A and H2B monoubiquitination was coincident with transcription, but not due to pol II catalyzed gene expression alone because anti-mUb-H2A and mUb-H2B ChIPs failed to enrich for the highly expressed Ramos β actin locus or the TPI gene in primary mouse splenocytes, even though pol II ChIP enriched for these regions at levels comparable to the re-*V_H_*, *c-MYC* Exons 1–3, and 17.2.25 (Figs. 3, 4). Furthermore, the only genomic regions enriched by mUb-H2A and mUb-H2B ChIP were those that were also enriched upon anti-AID ChIP (Fig. 3A); and these same loci also showed significant enrichments after ChIP with antibodies to the DNA repair factors PCNA and pol eta (Fig. S1), which are two proteins involved in somatic hypermutation opposite A and T bases (recently reviewed by [11]). Histone monoubiquitination may occur in response to AID activity, but could also be a signal for AID recruitment because AID colocalized with mUb-H2B as visualized by immunofluorescence microscopy (Fig. 5). Either way, our results identify the presence of both histone H2A and H2B tail monoubiquitinations at genes that are targeted selectively for mutagenesis, identifying this specific set of chromatin modifications with genomic loci that are deaminated by AID and mutated by responding DNA repair pathways. As such, co-occupancy of monoubiquitinated H2A and H2B within nucleosomes at the Ig genes may in part characterize the chromatin architecture associated with somatic hypermutation.

Histone termini extend away from the core histone octamer as tails that serve as acceptors for a wide range of post-transcriptional modifications. The combination of these modifications or “histone code” regulates a wide range of functions, although the specific DNA level activities triggered by various combinatorial signals have not been completely defined. In hypermutation, some small molecule histone modifications have been described. Acetylation of histone H3 and H4 at the IgH variable region, but not the constant region, results from activation of hypermutation in BL2 cells, suggesting a role in targeting somatic hypermutation machinery [34]. However, in primary mouse B cells changes in histone acetylation at hypermutating loci did not follow with B cell activation [35]. Nevertheless, H4 acetylation may be important for some types of AID-initiated diversification because disruption of the E2A transcription factor in DT40 leads to decreases in H4 acetylation and gene conversion [36]. One unique signal for V region hypermutation may be phosphorylation of histone H2A at serine 14 [35], and it is possible that this signal may operate in concert or in response to histone monoubiquitination to promote hypermutation. More than likely, locus-specific mutagenesis in B cells depends upon the combinatorial signal generated through multiple histone tail modifications, and further studies are required to decipher the specific histone code assigned to somatic hypermutation.

H2A and H2B monoubiquitination may have active roles in the hypermutation pathway, and immunofluorescence results confirm proximity of AID and monoubiquitinated H2B (Figs. 5, S5). Chromatin modification may occur as a consequence of AID activity to promote mutagenesis at some loci, or histone monoubiquitinations could help recruit AID. These models are not necessarily mutually exclusive. If chromatin ubiquitination is important for regulating the balance between mutagenic and faithful DNA repair in Ig gene hypermutation, one might predict that AID could deaminate multiple genes but the resulting uracils are faithfully repaired at the non-Ig loci. Indeed, we and others have observed multiple AID foci by immunofluorescence microscopy (Figs. 5, S5) [31]–[33], and genome sequencing of germinal center B cells reveals that AID acts widely at transcribed genes, but genomic instability is largely suppressed by faithful DNA repair [12]. There are likely to be signals specific to only some genes (like the Ig loci) that regulate the switch from faithful to mutagenic uracil repair, and because mUb-H2B appears to occupy only a small subset of the AID-targeted loci (Figs. 5, S5), chromatin ubiquitination is not an automatic result of AID binding but rather a locus-specific event. It is conceivable that the conspicuous presence of ubiquitinated H2A and H2B at the Ig genes represents a signal that marks these loci for mutagenesis.

Consistent with a model whereby histone monoubiquitination facilitates the repair responses to DNA deamination, recent studies have identified histone-specific E3 ubiquitin ligases in class switch recombination. Starting with histone H2A modification, ubiquitination cascades appear critical for establishing assembly of recombination factors at sites of AID activity. The loss of the RNF168 H2A E3 ligase disrupts histone ubiquitination and causes DNA repair defects and immunodeficiencies associated with RIDDLE syndrome [20]; and RNF8 works in concert with RNF168 to initiate and expand histone ubiquitinations at the Ig loci that resulting in 53BP1 activity and proper class switching [20]–[22]. In normal break repair, chromatin ubiquitinations promote assembly of DNA damage response factors at sites of ionizing radiation damage [37]. Furthermore, the replication cofactor PCNA, which we found physically associated with the re-*V_H_* (Fig. S1) and may be a lynchpin for regulating A/T biased hypermutation [11], colocalizes with monoubiquitinated H2A [38]. Upon AID activity, chromatin ubiquitination may signal assembly of hypermutation factors and/or ubiquitination cascades that regulate the shift between faithful uracil repair and mutagenesis. However, further studies are required in order to identify the ubiquitin E3 ligases functioning in normal and aberrant somatic hypermutation.

Histone monoubiquitination is a substantial chromatin modification, and suggests the DNA at AID-targeted loci is accessible for metabolism. Unlike small molecule modifiers like phosphates or acetyl groups, ubiquitin is an 11 kDa protein. The addition of a single ubiquitin on both H2A and H2B would increase nucleosome mass by nearly 40%, which may greatly influence the association between DNA and the core histones or create an assembly site for repair factors. The significant enrichments we obtained by ChIP of the re-*V_H_* and *MYC_14_* with mUb-H2A and H2B antibodies (Figs. 3B, C) suggest the presence of a highly ubiquitinated locus, with multiple antibody binding targets at the site of hypermutation, but apparently not extending into the IgH constant region (*V_H_1* amplicon, Figs. 3B, C). Thus, histone monoubiquitination is localized to the DNA regions that are also bound by AID. It follows that *V_H_1*, and likely the proximal S region, were not enriched by AID ChIP (Figs. 3A–C), which is expected because Ramos does not class switch. Because AID is active on single-stranded DNA and associated with the re-*V_H_* (Fig. 3A), it is conceivable that histone monoubiquitination creates a chromatin architecture that is permissible to AID attack or downstream uracil repair. Indeed, nucleosomal DNA is protected from AID in the absence of transcription [39], suggesting that deamination by AID may require histone remodeling and chromatin-level signaling. Importantly, our results identify a new chromatin modification associated with hypermutation and future studies will be required to next define functional roles in mechanisms of somatic hypermutation.

## Materials and Methods

### Cells and Cell lines

Ramos cells were purchased from the ATCC and were cultured in suspension at 37°C in RPMI (10% (v/v) FBS, 1% (v/v) PS, 2 mM L-glutamine) (Life Technologies, Grand Island, NY) in 75 cm^2^ flasks. Following limiting dilution cloning, a sIgM positive isolate was expanded for 90 days, and then analyzed for sIgM loss by flow cytometry. Ramos cells were collected by centrifugation at 300×g for 10 minutes, washed once with ice-cold PBS, and then resuspended in 100 µL PBS and incubated with 10 µL of anti-human-IgM antibody (Sigma, St Louis, MO) for 10 minutes at 4°C. Cells were again collected, washed twice with PBS, resuspended in 100 µL PBS and incubated with 3 µL of FITC-conjugated anti-goat Ig secondary for 10 minutes at 4°C in the dark. Cells were washed twice with PBS then resuspended in 400 µL PBS. Samples included 10% normal rat serum to prevent non-specific binding. Cells were analyzed immediately on a Becton Dickinson FACS Calibur flow cytometer using CellQuest Pro software (San Jose, CA) with 1×10^4^ events collected.

### Chromatin immunoprecipitations

Approximately 1×10^7^ cells were transferred to a 15-ml Falcon tubes and incubated for 10 minutes with 1% (v/v) formaldehyde. Chromatin was sheared by sonication to generate fragments averaging 500 bps, as judged by agarose gel electrophoresis. ChIPs were performed on prepared chromatin using EZ-ChIP kit reagent buffers (Millipore, Temecula, CA, 17–371) and standard manufacturer protocol. ChIP primary antibodies were as follows: normal mouse IgG negative control (Santa Cruz Biotechnology, Santa Cruz, CA, sc-2025), anti–RNA polymerase II (Millipore, 05-623B), anti-mUb-H2A (Millipore, 05-678) [41], anti-ub-H2B (Millipore, 05-1312) [42], anti-AID (Santa Cruz, sc-25620), and polyspecific IgG (Santa Cruz, sc-2025). Crosslinks were reversed by incubating chromatin at 65°C overnight, and enriched DNA template analyzed by traditional PCR and quantified by real-time PCR (described in PCR analyses).

### Western Blotting

Ramos cells at ∼1×10^6^ cells/ml, were collected by centrifugation, existing media removed, and cells resuspended in SDS lysis buffer containing protease inhibitors, and transferred to 1.5 ml Eppendorf tubes. Proteins were electrophoresed through a 4–12% SDS–polyacrylamide gradient gel (Invitrogen) and transferred to immobilon-P PVDF membranes (Millipore). Membranes were blocked for 1 hour in 5% (w/v) nonfat milk in phosphate-buffered saline containing 0.05% Tween 20, washed, and incubated with primary antibody overnight at 4°C using the following dilution: anti-mUb-H2A (Millipore, 05-678) –1∶500, anti-ub-H2B (Millipore, 05-1312) – 1∶500, and anti-AID (Santa Cruz, sc-25620) - 1∶1000. Membranes were washed and incubated with secondary Abs: HRP conjugated goat anti-mouse and goat anti-rabbit (Invitrogen) at 1∶10000 dilution. Immunoreactive bands were visualized with ECL Plus (Amersham, Piscataway, NJ) and signals were detected by using the Storm 840 PhosphorImager and IMAGEQUANT software (GE Healthcare Life Sciences).

### PCR analyses

Oligonucleotide sequences from all analyses are detailed in Table S1). Sequencing of Ramos *c-MYC* was performed as described previously [25]. Briefly, total Ramos RNA was converted to cDNA via RT-PCRs using random 20-mers and Protoscript Reverse Transcriptase (New England BioLabs (NEB), Ipswich, MA). Unless otherwise indicated, PCR amplifications were performed in 40 µl reactions at standard concentrations (1.5 mM MgCl_2_, 0.2 mM dNTP, 1× NEB PCR buffer, 0.5 U Taq (NEB), 0.5 uM each primer) and then cloned into Topo TA PCR 2.1 (Invitrogen, Carlsbad, CA, K450001) for sequencing. *MYC_14_* was distinguished from *c-MYC* on chromosome 8 by the presence of an A at position 523 to T polymorphism found within *MYC_14_* [25]. Traditional ChIP amplifications from recovered DNA was quantified by standard SybrGreen quantification (Invitrogen), then diluted to 0.1 ng/µl. PCR reactions were performed for 25, 30 or 35 cycles (as described above). Starting material was standardized across all reactions (0.1 ng eluted DNA), excluding inputs, which were titrated from 0.5 ng to 10 ng per reaction. Following amplification, products were resolved by 1.5% agarose gel electrophoresis. The identities of all amplicons were verified by Topo TA PCR 2.1 cloning and sequencing. Quantitative PCR (qPCR) used a series of primer pairs generated for each genomic locus using the Integrated DNA Technology design tool, PrimerQuest (https://www.idtdna.com). Individual amplicons were each evaluated by standard PCR ethidium bromide visualization, dissociation curve determination, and direct sequencing. A final validated primer pair was then selected for each locus (Table S1). Recovered DNA was diluted 1∶100 with ddH20 and 20 µl reactions prepared using DyNAmo SYBR Green qPCR 2× master mix (NEB, F400L) and ROX Reference Dye for Quantitative PCR (Sigma, R4526) were assembled in triplicate. Reactions were performed using an Applied Biosystems 7300 Real-Time PCR thermocycler (ABI, Foster City, CA) and DNA enrichments calculated by standard delta-delta Ct.

### Quasi-monoclonal mouse primary B cells

Spleens from six healthy quasi-monoclonal mice (created by [29]) were removed aseptically, placed in PBS and gently ground between frosted slides to produce a single-cell suspension. The suspension was centrifuged at 300×g for 5 minutes, the pellet was resuspended in ammonium-tris-chloride buffer to hypotonically lyse erythrocytes, and the remaining cells were washed with PBS by centrifugation at 300×g for 5 minutes. Splenocytes were cultured at 37°C/5% CO_2_/95% humidity in 75 cm^2^ flasks in RPMI 1640 (Life Technologies) supplemented with 100 U/ml of penicillin, 100 mg/ml of streptomycin and 10% FBS. Cultures were allowed to recover for 1 hour then supplemented with 25 µg/ml LPS (Sigma, L2143) and incubated for 72 hours to induce proliferation. Active Ig gene diversification was monitored by ELISA of the RPMI media for the presence of IgG, a result of LPS induced class switch recombination, as described [40], [43] and by ChIPs to show AID association with the 17.2.25 locus (Figure 4B). Cells were collected after 72 hours and immediately crosslinked with formaldehyde for ChIP analysis as described.

### Confocal immunofluorescence microscopy

Hypermutating Ramos cells were cultured in suspension at 37°C in RPMI (10% (v/v) FBS, 1% (v/v) PS, 2 mM L-glutamine) in 75 cm**^2^**flasks. At **∼**1×10^6^ cells/ml, 750 µL of Ramos suspensions were transferred into individual poly-lysine/ConA-coated chambered slide wells and incubated at 37°C for 1 hour to allow adhesion. Cells were rinsed twice with PBS then fixed with 2% paraformaldehyde for 20 minutes. Cells were again rinsed twice with PBS then blocked with for 30 minutes (PBS, 1% Goat serum, 0.2% TritonX-100). Cells were next incubated with primary antibodies diluted 1∶1000 in PBTG (PBS, 1% Goat serum, 0.2% TritonX-100) for 2 hours at room temperature. Cells were rinsed 3 times with PBTG then incubated in the dark for 2 hours at room temperature with secondary antibodies diluted 1∶1000 in PBTG. Cells were again rinsed 3 times with PBTG then mounted with vectashield (Vector Laboratories, Burlingame, CA, H1000). Confocal images were obtained using a Leica TCS SP2 Confocal Microscope and contrast processed with Adobe Photoshop 7.0 software. Primary antibodies included: anti-mUb-H2B (Millipore, no. 05-1312) and anti-AID (Santa Cruz, sc-25620). Anti-mUb-H2A (Millipore, 05-678) was also examined but produced no discernable immunofluorescence microscopy signal. Secondary Abs were goat anti-mouse conjugated to Alexa 555 and goat anti-rabbit conjugated to Alexa 488 (1∶1000) (Invitrogen). DAPI (1∶1000) Alexa 633-wheat germ agglutinin (1∶1000) was used to stain nuclear envelopes (Invitrogen).

## Supporting Information

Figure S1 PCNA and polymerase eta are associated with the Ramos re-VH region. Ramos cells were crosslinked, and sheared chromatin subjected to immunoprecipitation using antibody specific to PCNA, MSH2 and polymerase eta, and non-specific IgG antibody. Precipitated template DNA was amplified by qPCR using primers specific to a control glutamine tRNA locus (tRNA), unrearranged VH (V8), and the rearranged VH (VH2)(see Figure 1). Enrichment using poly-specific IgG for each locus was subtracted to normalize for non-specific IP. Fold enrichment relative to input DNA is shown.
(3.87 MB TIF)

Figure S2 HEK293 and A549 ChIPs using AID, mUb-H2A and mUb-H2B antibodies find no enrichment of sequences undergoing hypermutation in Ramos cells. Normalized template enrichment upon anti-AID, anti- mUb-H2A and anti-mUb-H2B ChIPs. Template obtained from IPs was amplified and analyzed by qPCR using primer sets diagramed in Figure 1. Results were normalized to non-specific IgG IP and relative to input. Each bar indicates the mean of the values obtained in triplicate with standard deviation.
(2.57 MB TIF)

Figure S3 AID antibody used for ChIPs is specific for AID. Western blot analysis of endogenous AID (24 kDa) in AID positive (Ramos) and in AID negative (HEK293) cell lysates. PCNA was utilized as load control.
(5.65 MB TIF)

Figure S4 AID IP demonstrating AID antibody used for ChIPs is detecting AID. Western blot analysis of endogenous AID (24 kDa) in Ramos cell lysate IP. Primary anti-AID for IP, (Santa Cruz Biotechnology, Santa Cruz, CA sc-25620). Primary Ab for western, (Santa Cruz Biotechnology, Santa Cruz, CA sc-14680). Total supernatant and total IP were loaded to ensure equivalent starting material.
(3.64 MB TIF)

Figure S5 mUb-H2b colocalizes with AID in multiple discrete foci. Representative immunofluorescence microscopy images of two distinct Ramos cells stained with AID and mUb-H2B antibodies and exhibiting colocalization of these at 1 (Panel A) and 2 (Panel B) discrete foci. In each panel: Upper left, Ramos cells imaged with Alexa 488 filter. Upper right, Ramos cells imaged with Alexa 555 filter. Lower left, merged images with white scale bar (2 microns). Lower right nuclear envelope imaged using wheat germ agglutinin (Blue). Arrows point to colocalizations of AID and mUb-H2B.
(9.14 MB TIF)

Table S1 Oligonucleotide table. Oligonucleotide sequences for amplification of hypermutating genomic and control loci in this study are shown.
(0.06 MB DOC)
