# Supplementary figures and images for: Histone H2A and H2B Are Monoubiquitinated at AID-Targeted Loci
Source: PLoS One. 2010 Jul 16;5(7):e11641. doi: 10.1371/journal.pone.0011641 (PMC2905439; doi:10.1371/journal.pone.0011641)

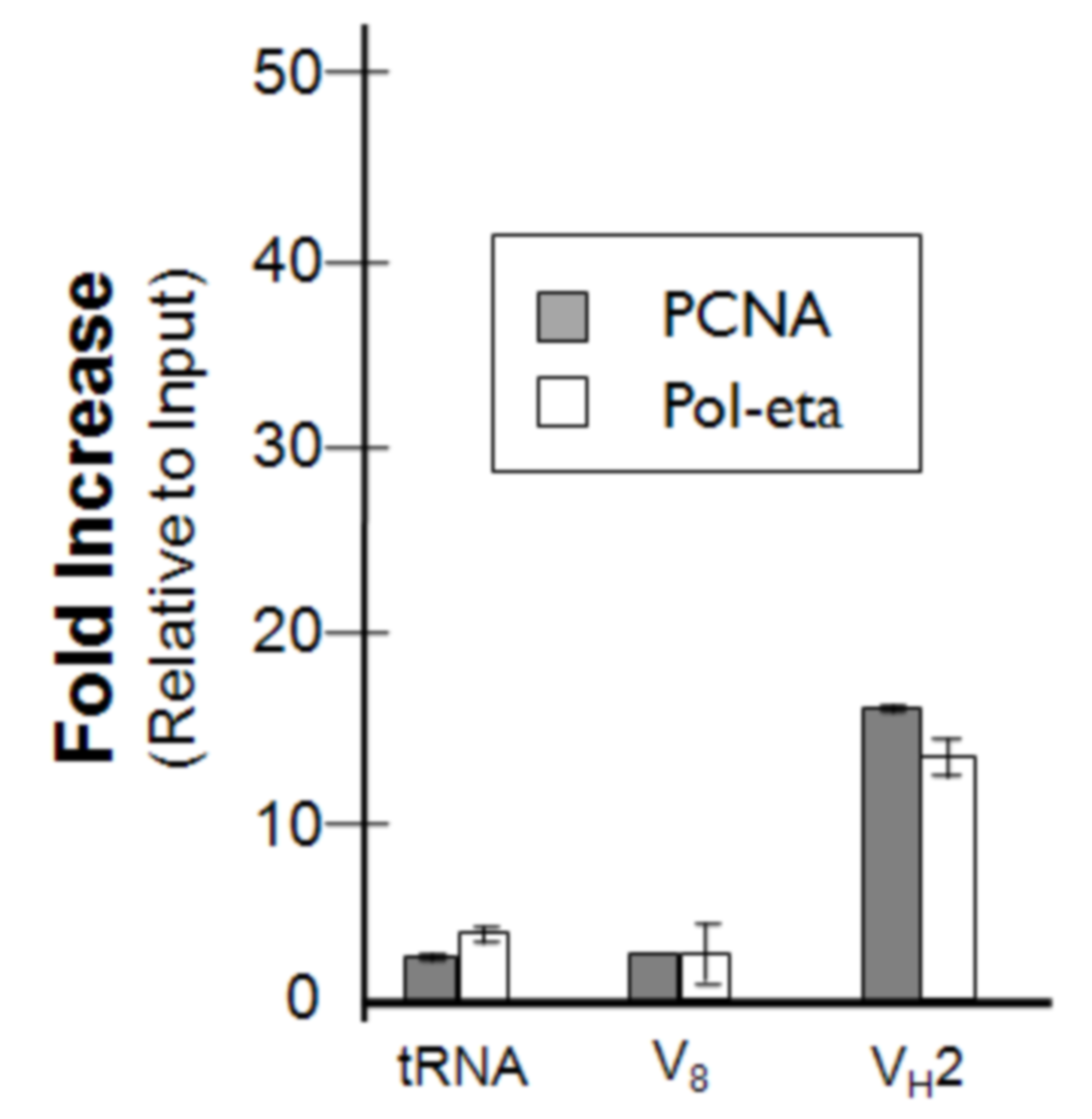

Supplement: Figure S1 — PCNA and polymerase eta are associated with the Ramos re-VH region. Ramos cells were crosslinked, and sheared chromatin subjected to immunoprecipitation using antibody specific to PCNA, MSH2 and polymerase eta, and non-specific IgG antibody. Precipitated template DNA was amplified by qPCR using primers specific to a control glutamine tRNA locus (tRNA), unrearranged VH (V8), and the rearranged VH (VH2)(see Figure 1). Enrichment using poly-specific IgG for each locus was subtracted to normalize for non-specific IP. Fold enrichment relative to input DNA is shown. (3.87 MB TIF) [file pone.0011641.s001.tif]

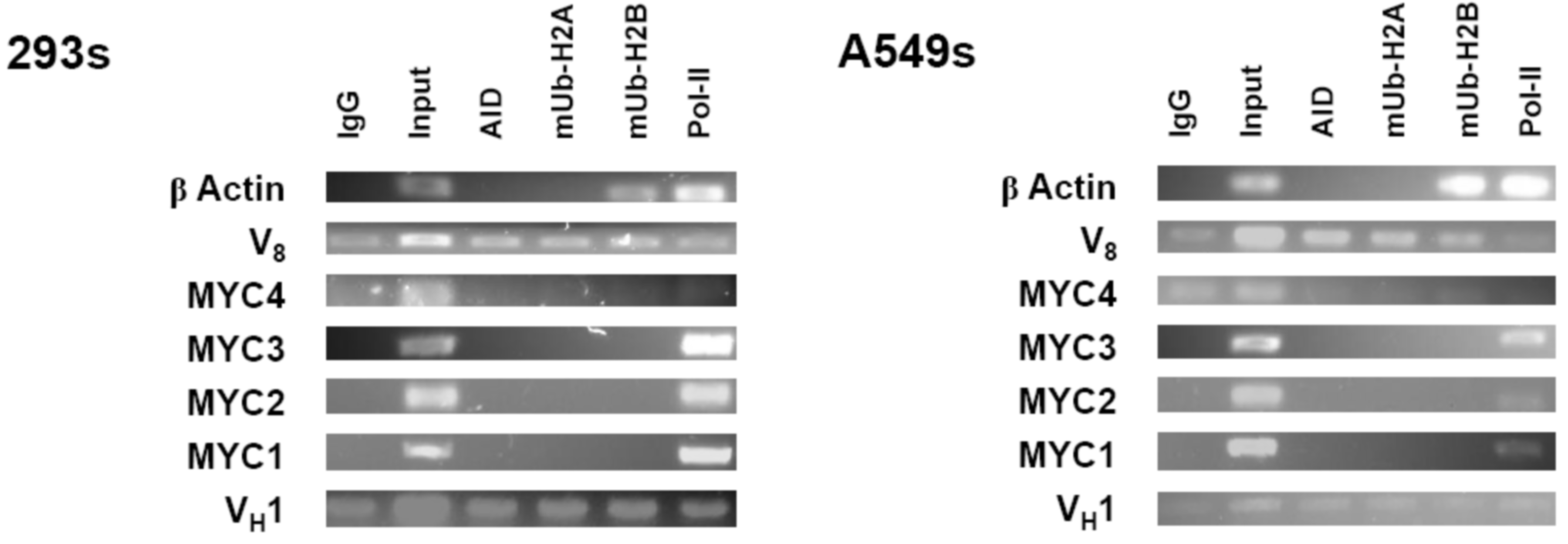

Supplement: Figure S2 — HEK293 and A549 ChIPs using AID, mUb-H2A and mUb-H2B antibodies find no enrichment of sequences undergoing hypermutation in Ramos cells. Normalized template enrichment upon anti-AID, anti- mUb-H2A and anti-mUb-H2B ChIPs. Template obtained from IPs was amplified and analyzed by qPCR using primer sets diagramed in Figure 1. Results were normalized to non-specific IgG IP and relative to input. Each bar indicates the mean of the values obtained in triplicate with standard deviation. (2.57 MB TIF) [file pone.0011641.s002.tif]

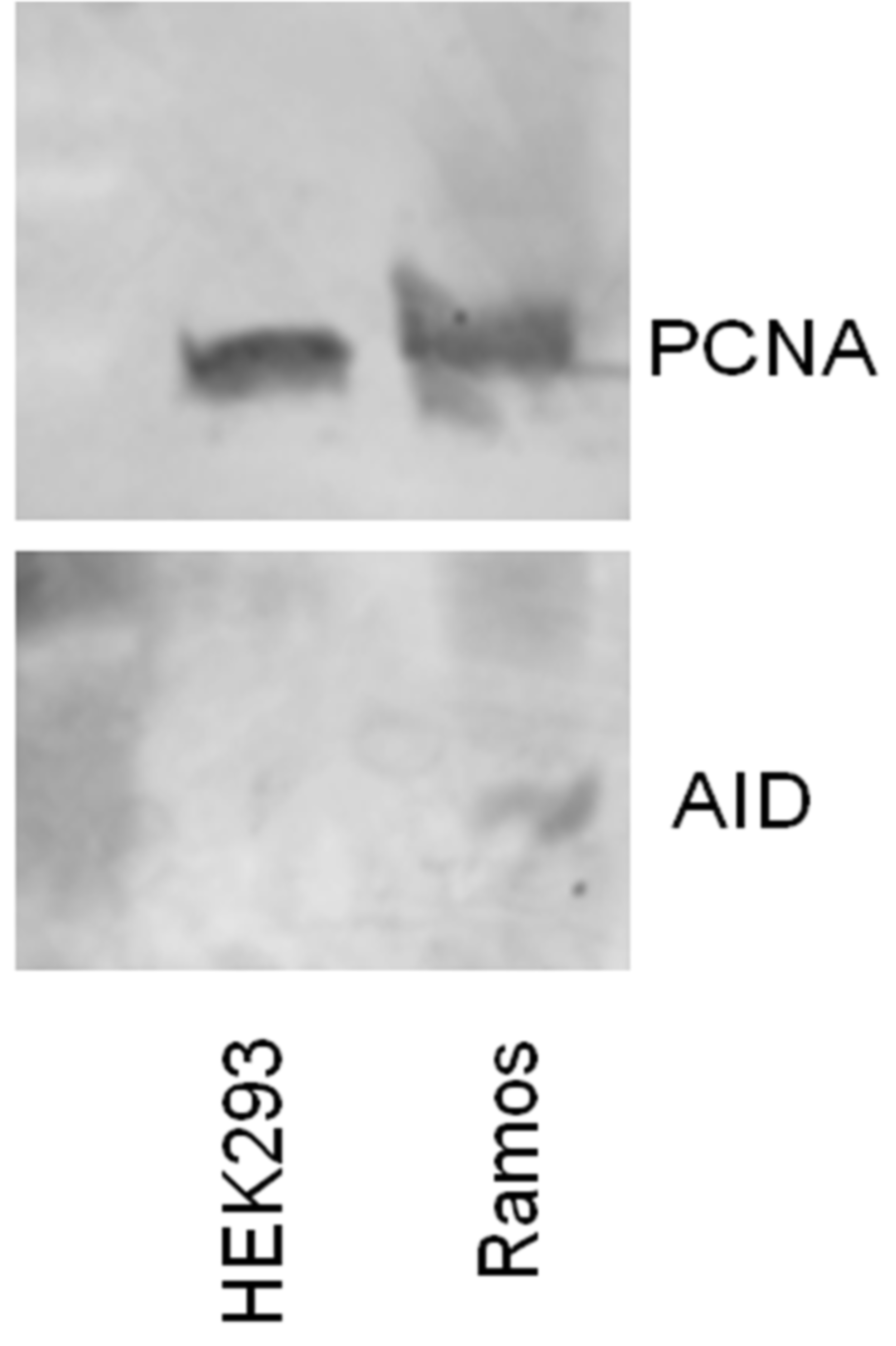

Supplement: Figure S3 — AID antibody used for ChIPs is specific for AID. Western blot analysis of endogenous AID (24 kDa) in AID positive (Ramos) and in AID negative (HEK293) cell lysates. PCNA was utilized as load control. (5.65 MB TIF) [file pone.0011641.s003.tif]

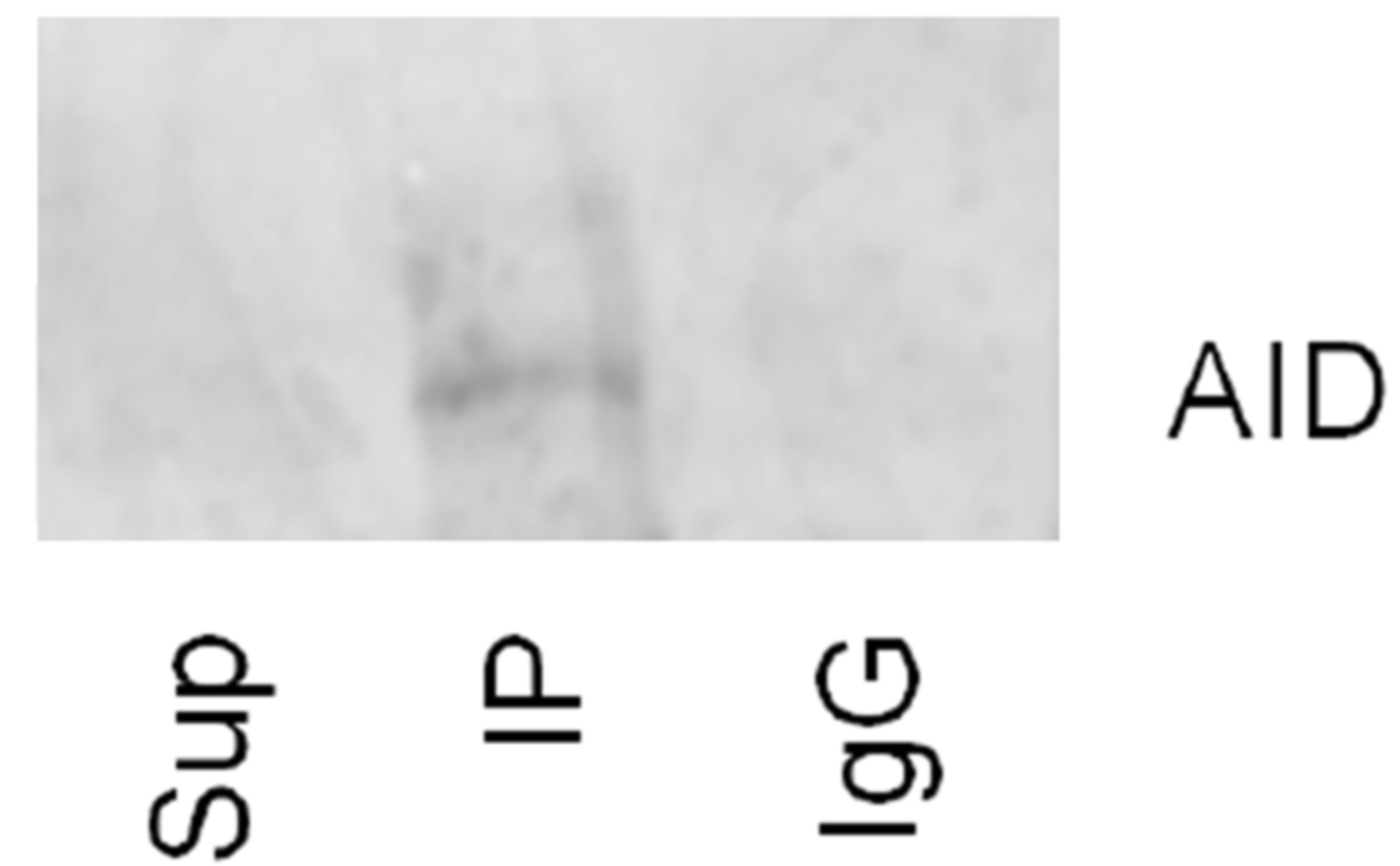

Supplement: Figure S4 — AID IP demonstrating AID antibody used for ChIPs is detecting AID. Western blot analysis of endogenous AID (24 kDa) in Ramos cell lysate IP. Primary anti-AID for IP, (Santa Cruz Biotechnology, Santa Cruz, CA sc-25620). Primary Ab for western, (Santa Cruz Biotechnology, Santa Cruz, CA sc-14680). Total supernatant and total IP were loaded to ensure equivalent starting material. (3.64 MB TIF) [file pone.0011641.s004.tif]

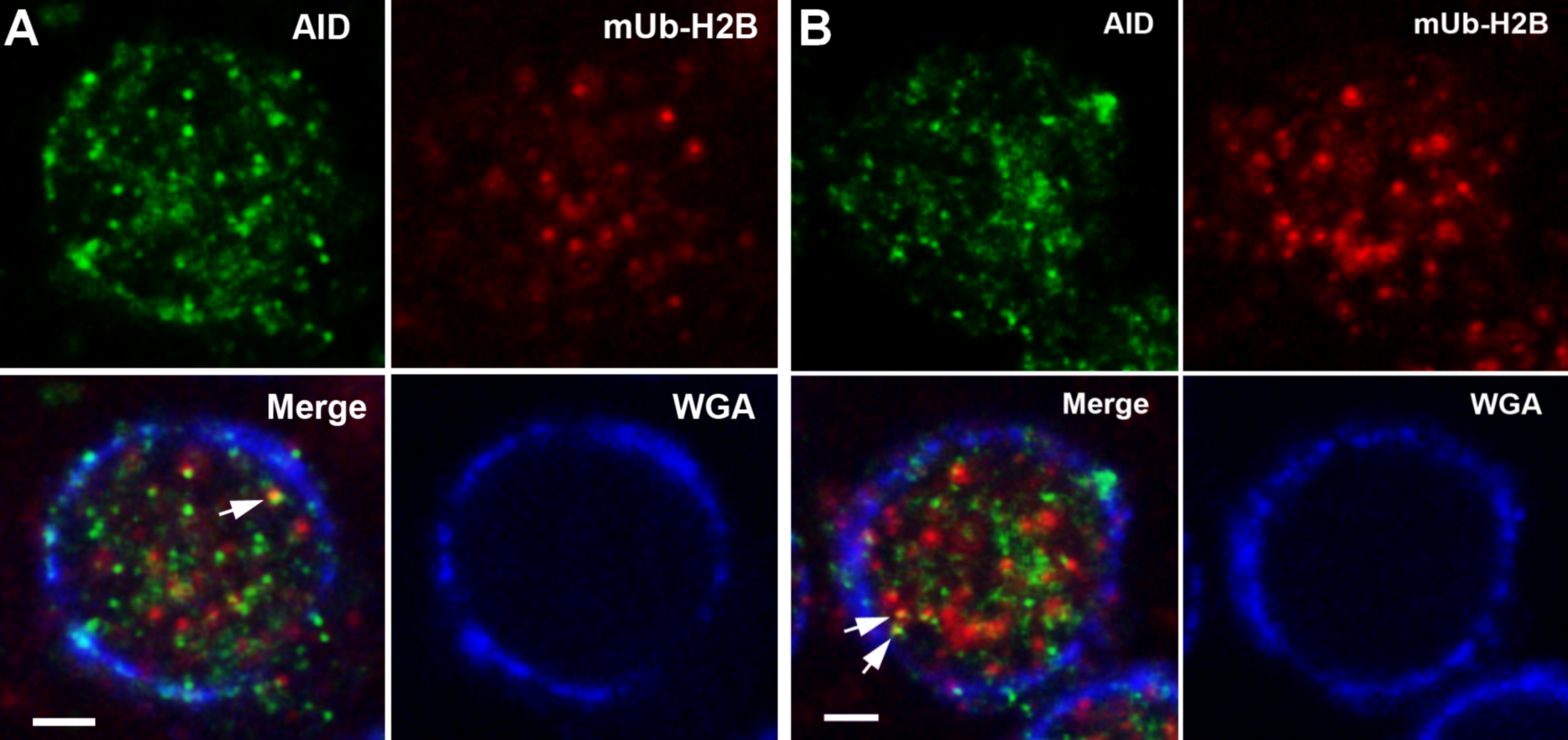

Supplement: Figure S5 — mUb-H2b colocalizes with AID in multiple discrete foci. Representative immunofluorescence microscopy images of two distinct Ramos cells stained with AID and mUb-H2B antibodies and exhibiting colocalization of these at 1 (Panel A) and 2 (Panel B) discrete foci. In each panel: Upper left, Ramos cells imaged with Alexa 488 filter. Upper right, Ramos cells imaged with Alexa 555 filter. Lower left, merged images with white scale bar (2 microns). Lower right nuclear envelope imaged using wheat germ agglutinin (Blue). Arrows point to colocalizations of AID and mUb-H2B. (9.14 MB TIF) [file pone.0011641.s005.tif]
