# Supplementary material for: Histone H2A and H2B Are Monoubiquitinated at AID-Targeted Loci
Source: PLoS One. 2010 Jul 16;5(7):e11641. doi: 10.1371/journal.pone.0011641 (PMC2905439; doi:10.1371/journal.pone.0011641)
Supplement: Table S1 — Oligonucleotide table. Oligonucleotide sequences for amplification of hypermutating genomic and control loci in this study are shown. (0.06 MB DOC) [file pone.0011641.s006.doc]

**Human QPCR Primers**

**V_H_1**

V_H_1F CCCAAGGACACCCTCATGATCTCCC

V_H_1R CATTATGCACCTCCACGCCGTCCAC

**V_H_2**

V_H_2/8F GCAGGACTGTTGAAGCCTTCGGAG

V_H_2R CTTACCTGAGGAGACGGTGACC

**V_H_3**

V_H_3F GACTAACCGGAGTTTCATGGCTGTGTTG

V_H_3R GAGGTCTCAAACTTGATGGTTTGCAGATG

**V8**

V_H_2/8F GCAGGACTGTTGAAGCCTTCGGAG

V8R TGCAGGGAGGTTTTTGTCTGGG

**MYC1**

MYC1F CCTCTCTCGCTAATCTCCGCCC

MYC1R CGCTCGCTCCCTCTGCCTCTC

**MYC2**

MYC2F CTGTATGTGGAGCGGCTTCTCG

MYC2R CTGCTGTCGTTGAGAGGGTAG

**MYC3**

MYC3F CCACAGCAAACCTCCTCACAGCCCAC

MYC3R CTCTGACACTGTCCAACTTGACCCTC

**MYC4**

MYC4F TCACCATCTTGACTCCTACCTTAGCC

MYC4R CTGAACTGGCTTCTTCCCAGGAGCC

**β Actin**

bActF GGCTCCGGCATGTGCAAGG

bActR GGTGAGGATGCCTCTCTTGCTCTG

**tRNA^glu^**

trna_glu_F AAGGCGCATCTCTAGTTCAGTGG

trna_glu_R2 CTCTTTGCTCCTGGAGTCTCTCAC

**Mouse QPCR Primers**

**STEP**

F2_neural_59.7 CCAGAGGACAGGCGTCAATCAG

R1_neural_59.9 GTCAATGAGGCAGGATCAAGTCTCAGG

**TPI**

mus_tpi_2F61 CCTCTAGAACATTGATTGCTCTAGAACGCC

mus_tpi_R61 TCAGTACAAACGCACGGCTCTCTC

**17.2.25**

F4_17.2.25_59 CTACAGGGGTCAATTCAGAGGTTCAG
R1_17.2.25_63 GGCAGTGTCCTCAGATGTCAGGC

**Sγ3**

Sγ3F AGTCACCATGTAGATACTCAGCCTG

Sγ3R CAGGTTATGCAGCTCCTGGGTCAG

**MYC MUTANT ANALYSIS**

**Exon1**

MYC1F CCTCTCTCGCTAATCTCCGCCC

X1mutR CACCATCTCCAGCTGGTCGGCC

**Exon2**

MYC2F CTGTATGTGGAGCGGCTTCTCG

MYC3R CTCTGACACTGTCCAACTTGACCCTC

**Exon3**

X3mutF CCACAGCATACATCCTGTCCGTCC

X3mutR CCTACCTCTCACCTTCTCACCTGCC
